# Axillary nerve conduction changes in hemiplegia

**DOI:** 10.1186/1749-7221-3-26

**Published:** 2008-12-17

**Authors:** Atzmon Tsur, Haim Ring

**Affiliations:** 1 Rehabilitation Department, Western Galilee Hospital, POB 21, Nahariya, Israel; 2 Loewenstein Rehabilitation Hospital, POB 3, Ra'anana and Sackler Faculty of Medicine, Tel-Aviv University, Tel Aviv-Yafo, Israel

## Abstract

**Aim:**

To prove the possibility of axillary nerve conduction changes following shoulder subluxation due to hemiplegia, in order to investigate the usefulness of screening nerve conduction studies in patients with hemiplegia for finding peripheral neuropathy.

**Methods:**

Forty-four shoulders of twenty-two patients with a first-time stroke having flaccid hemiplegia were tested, 43 ± 12 days after stroke onset. Wasting and weakness of the deltoid were present in the involved side. Motor nerve conduction latency and compound muscle action potential (CMAP) amplitude were measured along the axillary nerve, comparing the paralyzed to the sound shoulder. The stimulation was done at the Erb's point whilst the recording needle electrode was inserted into the deltoid muscle 4 cm directly beneath the lateral border of the acromion. Wilcoxon signed rank test was used to compare the motor conduction between the sound and the paralytic shoulder. Mann-Whitney test was used to compare between plegic and sound shoulder in each side.

**Results:**

Mean motor nerve conduction latency time to the deltoid muscle was 8.49, *SD *4.36 ms in the paralyzed shoulder and 5.17, *SD *1.35 ms in the sound shoulder (p < 0.001).

Mean compound muscle action potential (CMAP) amplitude was 2.83, SD 2.50 mV in the paralyzed shoulder and was 7.44, SD 5.47 mV in the sound shoulder (p < 0.001). Patients with right paralyzed shoulder compared to patients with right sound shoulder (*p *< 0.001, 1-sided for latency; *p *= 0.003, 1-sided for amplitude), and patients with left paralyzed shoulder compared to patients with left sound shoulder (*p *= 0.011, 1-sided for latency, p = 0.001, 1-sided for amplitude), support the same outcomes. The electro-physiological changes in the axillary nerve may appear during the first six weeks after stroke breakout.

**Conclusion:**

Continuous traction of the axillary nerve, as in hypotonic shoulder, may affect the electro-physiological properties of the nerve. It most probably results from subluxation of the head of the humerus, causing demyelinization and even axonopathy. Slowing of the conduction velocities of the axillary nerve in the paralyzed shoulders may be related also to the lowering of the skin temperature and muscular atrophy in the same limb. The usefulness of routine screening nerve conduction studies in the shoulder of hemiplegic patients seems to be advocated.

## Introduction

It is well known that shoulder subluxation in hemiplegics is one of the disabling factors encountered in rehabilitating patients. The causative factors may include the pull of gravity on the paralyzed shoulder [1], peripheral nerve lesions [2] or tear in the rotator cuff [3]. Hemiplegic extremities are usually recognized as being flaccid during the early stage following cerebrovascular accident, and this may cause migration of the humeral head in the shoulder joint leading to overstretching of the capsule, tendons and ligaments along with the brachial plexus [4-6]. The mechanism of the palsy appears to involve a stretch injury. The hemiplegic patient without complications most commonly shows a course in which flaccidity is followed by spasticity, and in which return of function and muscle tone proceeds from proximal to distal muscle groups [7,8].

An axillary nerve lesion caused by prolonged stretching, can be expressed by numbness over part of the outer shoulder, difficulty in lifting objects with the sore arm and in raising it above the head. These symptoms will blur the successful results of the rehabilitation after stroke if the axillary nerve is involved.

The aim of the study was to prove the probability of axillary nerve lesion after shoulder injury due to hemiplegia and so, to improve preventive and corrective measures for this difficult condition, knowing that even in case of complete recovery from hemiplegia, a disability will remain as a result of this lesion.

## Methods

**Figure 1:**
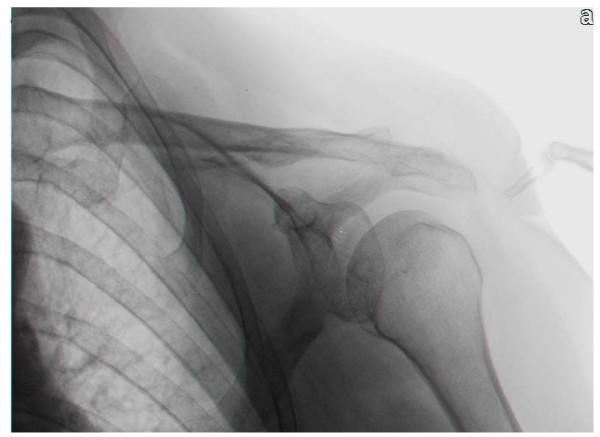
**One or more fingers breadths in the upper part of gleno-humeral joint space, between the acromion and the humeral head of the paralyzed shoulder**.

The study was a retrospective analysis of data on patients hospitalized in our rehabilitation department between the years 2003 and 2006. We routinely perform nerve conduction tests on all stroke patients who have flaccid paralysis in the upper limb [9]. Twenty-two inpatients suffered from hemiplegia after first-time stroke, included 8 men and 14 women, were tested. Their mean age was from 50 to 90 years (mean 72.5 ± 9.5 years) and the duration of the hemiplegia at the time of examination varied from 25 to 87 days (mean = 43 ± 12 days, and median = 43 days). Eleven patients had right hemiplegia and the remaining eleven, left hemiplegia. All patients were right hand dominant. The causes of hemiplegia were cerebral infarction in 16 patients, cerebral hemorrhage in 4 patients and cerebral hemorrhage inside infarction in 2 patients. Selection criteria were paralysis of upper limb after first-time stroke, flaccidity and atrophy of shoulder girdle muscles in the involved side and one or more fingers breadths in the upper part of the gleno-humeral joint space of the paralyzed shoulder (Figure 1). All patients had no previous history of trauma or peripheral nerve injury in the paralyzed upper extremities. All patients who had flaccid paralysis after a second or later stroke, were excluded from the study.

Nerve conduction studies were performed by the first author in a closed room in which the temperature was maintained at 22–24° Celsius, while the patient was placed in a sitting position, on his wheelchair, with the arm at 45 degrees abduction. All patients were studied on a Nicolet Viking III P, Madison Wisconsin, USA electromyography machine. Electrical nerve stimulation of 200 Volts, well tolerated by the patients, was given at the Erb's point, slightly above the upper margin of the clavicle and lateral to the clavicular head of the sternocleidomastoid muscle. Stimulator pulse duration of the square wave was 0.1 msec. A coaxial needle for registration was inserted into the middle deltoid muscle, 4 cm directly beneath the lateral border. The ground electrode was placed between the stimulating and the pick-up electrode [10]. The latency was measured from the stimulus artifact to the CAMP onset point and the amplitude was determined from baseline to the highest negative peak [11,12].

Results of the paralyzed shoulder were compared to those obtained in the sound shoulder.

We had to take into consideration that there was an asymmetry between the shoulders, due to muscular atrophy in the paralyzed side. Due to technical disorders, skin temperature was measured only in few patients.

### Statistical analysis

A descriptive statistical study of the quantitative parameters of mean and standard deviation was performed, and the Wilcoxon signed rank sum test was used to compare the quantitative data presented as latencies and amplitudes between the healthy and the paralyzed sides (assumption of normal distribution could not be held for differences). Additionally, 11 patients having right shoulder paralysis were compared with 11 patients having right healthy shoulders and separately, another 11 patients having left shoulder paralysis were compared with 11 having healthy left shoulders, using the Mann-Whitney test. P values below 0.05 were taken to indicate statistical significance. SPSS for Windows version 11.5 (Chicago, IL) was used for the statistical analysis.

## Results

The mean latency time to the deltoid was 8.49 ms, *SD *= 4.36 in the paralyzed shoulder and 5.17 ms, *SD *= 1.35 in the sound shoulder (Wilcoxon signed rank test, *p *< 0.001, 1-sided).

The mean compound muscle action potential (CMAP) amplitude was 2.83 mV, *SD *= 2.50 in the paralyzed shoulder and was 7.44 mV, *SD *= 5.47 in the sound shoulder (Wilcoxon signed rank test, *p* < 0.001, 1-sided), (Table 1).

**Table 1:** CMAP latency and amplitude recorded in the deltoid muscle

	Sound shoulder	Paralyzed shoulder	p-value
CMAP latency	5.17 ± 1.35	8.49 ± 4.36	< 0.001, 1-sided

CMAP amplitude	7.44 ± 5.47	2.83 ± 2.50	< 0.001, 1-sided

The same tendencies were found significant when this comparison was done separately for patients with a right paralyzed shoulder (N = 11) and for patients with left paralyzed shoulders (N = 11). Patients with right paralyzed shoulder compared to patients with right sound shoulder (*p *< 0.001, 1-sided for latency; *p *= 0.003, 1-sided for amplitude), and patients with left paralyzed shoulder compared to patients with left sound shoulder (*p *= 0.011, 1-sided for latency, *p *= 0.001, 1-sided for amplitude), support the same outcomes.

The mean latency time to the deltoid in patients tested up to 43 days after stroke breakout was 9.3 ms (*SD *= 4.55) in the paralyzed shoulder and 5.3 ms (*SD *= 1.5) in the sound shoulder (Wilcoxon signed rank test, *p *= 0.007, 1-sided). The mean CMAP amplitude in patients tested up to 43 days after stroke breakout was 2.8 mV, *SD *= 2.4 in the paralyzed shoulder and 6.5 mV, *SD *= 5.1 in the sound shoulder (Wilcoxon signed rank test, *p *= 0.001, 1-sided) (Table 2, Table 3).

**Table 2:** CMAP latency and amplitude recorded in the deltoid m. up to 43 days after stroke onset

	Sound shoulder	Paralyzed shoulder	p-value
CMAP latency	5.3 ± 1.5	9.3 ± 4.55	0.007, 1-sided

CMAP amplitude	6.5 ± 5.1	2.8 ± 2.4	0.001, 1-sided

**Table 3:** CMAP latency and amplitude recorded in the deltoid m. over 43 days after stroke onset

	Sound shoulder	Paralyzed shoulder	p-value
CMAP latency	5.07 ± 1.2	7.55 ± 4.15	0.02, 1-sided

CMAP amplitude	8.5 ± 5.98	2.88 ± 2.7	0.005, 1-sided

## Discussion

Electrophysiological investigations of shoulder subluxation in hemiplegic patients has been well documented in several reports [7,9,13,14]. Milanov [15] who evaluated the motor conduction in median, ulnar, peroneal and tibial nerves, found that the mean M-wave amplitudes were significantly decreased for each nerve study, in both upper and lower limbs of the paralyzed limbs, compared with the healthy side. In contrast, the mean motor conduction velocities were not reduced in the involved limbs compared to the unaffected limbs. Their patients were with long-term spastic hemiplegia after stroke. In our study, both the motor latency and the M-wave amplitude were significantly reduced in the paralyzed side, taking into consideration that our patients had in contrast, short-term flaccid hemiplegia.

The muscular tone in the paralyzed upper limb of our patients remained flaccid for more then several weeks. In the flaccid stage of stroke, the shoulder is prone to inferior subluxation and vulnerable to soft-tissue damage; weakness in the shoulder girdle muscles and gravitational pull tend to result in inferior subluxation [16-18].

Does a downward subluxation may produce traction on the axillary nerve as it winds around the surgical neck of the humeral shaft?

Injury to the axillary nerve in stroke patients may result from a traction force. In the present study, the latency time to the deltoid muscle showed delayed latency values and the CMAP amplitude showed reduced values in the axillary nerve on the paralyzed side.

There is sufficient biomechanical evidence that the peripheral nerve under tension undergoes strain and glides within its interfacing tissue [19]. The weight of the unsupported arm may also cause traction damage to various nerves including the axillary nerve [20], the suprascapular nerve [21] and the brachial plexus [1]. Ring et al [9] found that among 6 stroke patients that manifested certain deterioration of their gleno-humeral alignment, 5 had an electromyographic feature of axillary nerve damage. The most common zone of injury is just proximal to the quadrilateral space [22]. Ring et al [14] suggested that downward subluxation is able to produce traction on the axillary nerve as it winds around the surgical neck of the humeral shaft. The presence of an atypical pattern including flaccidity and atrophy of the supraspinatus, infraspinatus, deltoid and biceps muscles in the impaired upper extremity, in the presence of increased muscle tone or movement in the distal muscles, should alert caregivers to the possibility of complicating brachial plexus lesion [7].

We must also take into consideration that the prolonged latency registered after giving an electrical stimulation of the axillary nerve in the paralyzed shoulder, may be related also to the lowering of the skin temperature in the affected limbs. In chronic hemiplegia a decrease in temperature may result from inactivity of the limbs and reduced circulation [23].

Wasting of muscles in the shoulder girdle, among them the deltoid muscle, in patients after lesions of the upper motor neuron, can be a cause of reduced conduction velocity [24]. McComas et al [25] described a possible mechanism for muscle atrophy following upper motoneuron lesions. We believe that a decreased diameter of the nerve fiber as a result or cause of muscle atrophy, could lead to a decreased nerve conduction velocity.

We believe that continuous traction of the axillary nerve, as in the hypotonic shoulder, may affect the electro-physiological properties of the nerve. It most probably results from subluxtion of the head of the humerus, causing demyelinization and even axonopathy. Myelin loss results in slowing of the nerve conduction through the area involved. When traction is severe, an axonal damage, expressed by reduction of CMAP amplitude, may occur. We cannot disregard the fact that slowing of the conduction velocities of the axillary nerve in the paralyzed shoulders may be related also to the lowering of the skin temperature in the same limbs.

The difference between the mean latency time and CMAP amplitude in the paralyzed compared to the sound shoulder, tested up to 43 days after stroke breakout, was statistically significant. Rehabilitation of stroke patients with hemiplegia takes place generally in the first two or three months of the disease, meaning that the onset of axillary nerve lesion in the paralyzed side is early, and happens generally during the rehabilitation period.

Most stroke patients with hemiplegia will experience shoulder injury and pain [26]. Nerve lesions secondary to subluxation or dislocation may retard or be detrimental for muscle recovery and limb function [9].

## Conclusion

The initial flaccidity of the hemiplegic shoulder can result in the axillary nerve lesion associated with shoulder subluxation. It is advocated that electrophysiological studies of the shoulder girdle be carried out, several weeks after stroke breakout, to assess the severity of peripheral nerve involvement, so early preventive measures for shoulder subluxation and subsequent nerve damage can be applied. We are not able to propose the exact mechanism of lower motor neuron degeneration, but our findings are compatible with myelin changes in motoneurons followed by axonal involvement.

## Competing interests

The authors declare that they have no competing interests.

## Authors' contributions

AT performed all the examinations on the patients, wrote the manuscript and collected the references. HR proposed the initial design.
